# Supplementary figures and images for: Apolipoprotein-CIII O-Glycosylation, a Link between GALNT2 and Plasma Lipids
Source: Int J Mol Sci. 2023 Oct 2;24(19):14844. doi: 10.3390/ijms241914844 (PMC10573541; doi:10.3390/ijms241914844)

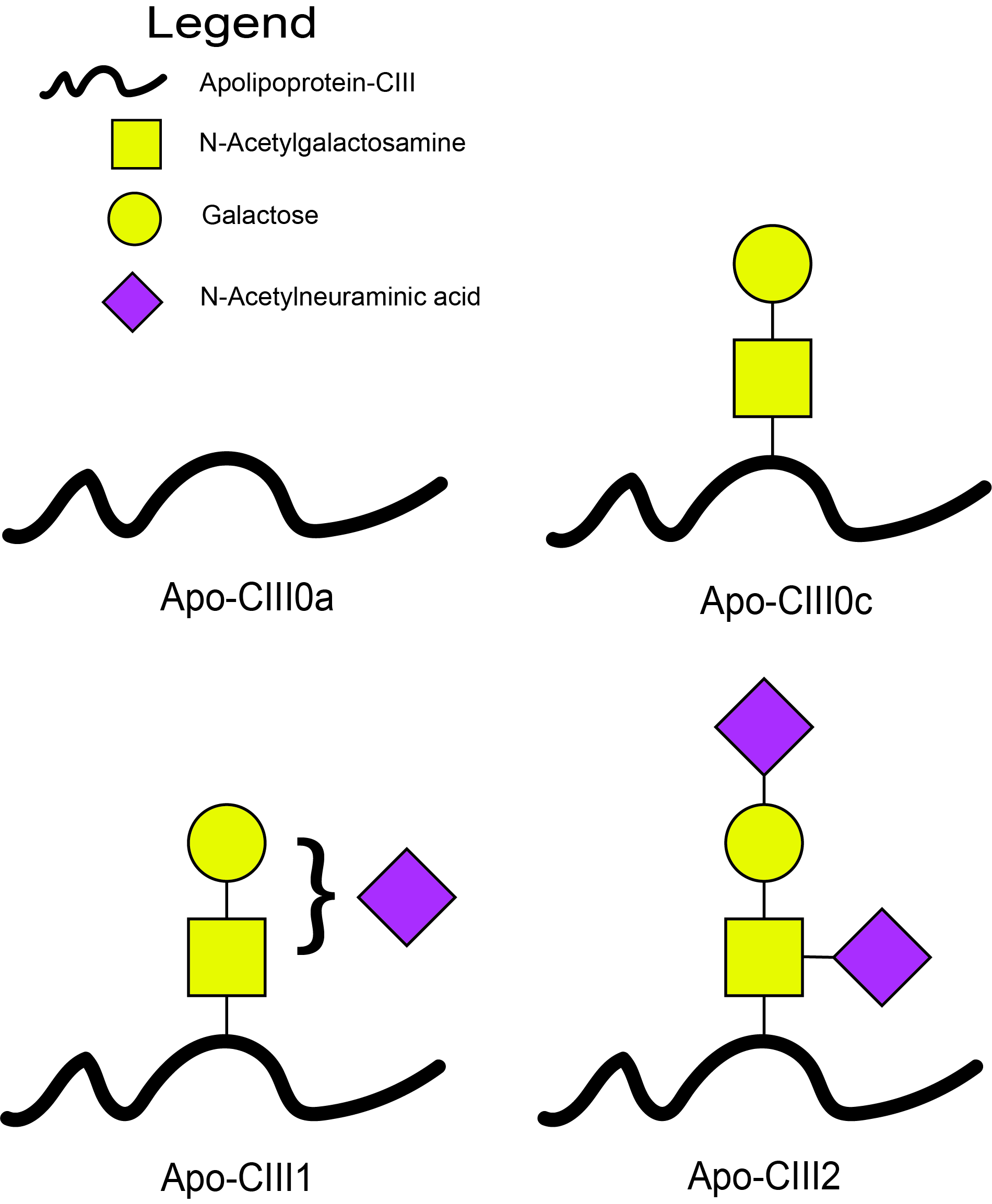

Supplement: Supplementary file 1 [file ijms-24-14844-s001.zip › Supplementary Figure S1new.tif]

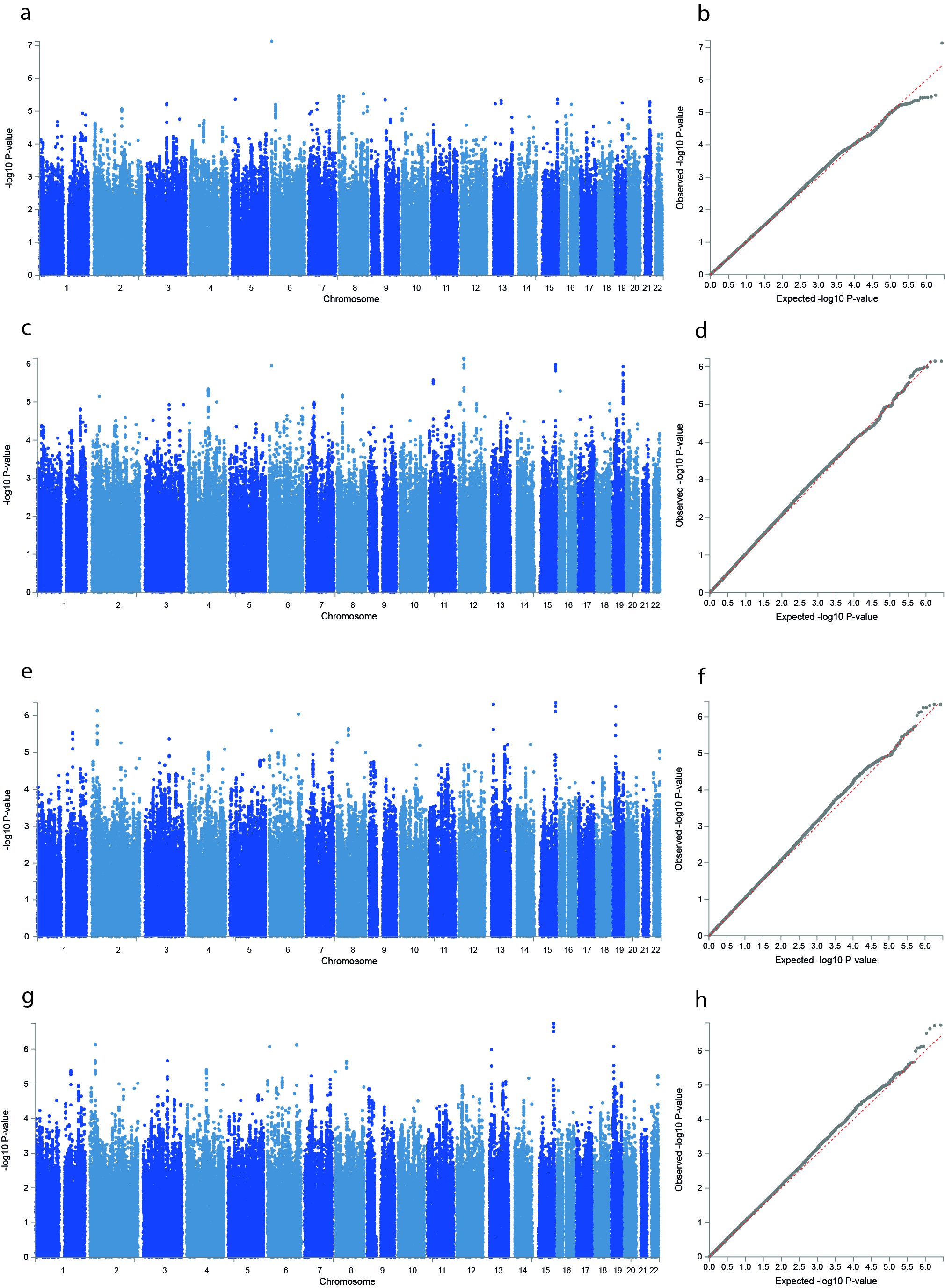

Supplement: Supplementary file 1 [file ijms-24-14844-s001.zip › Supplementary figure S2new.tif]

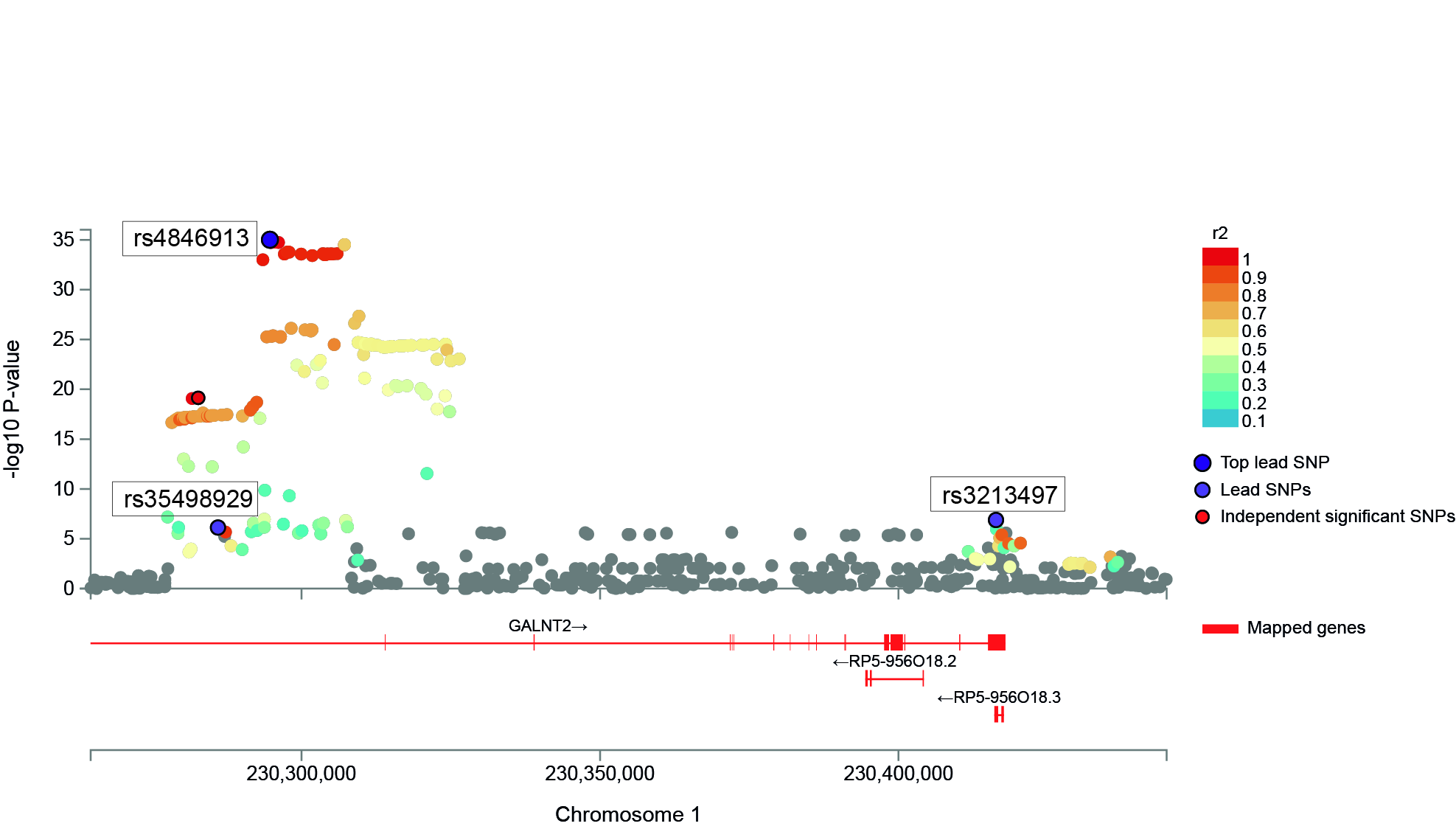

Supplement: Supplementary file 1 [file ijms-24-14844-s001.zip › Supplementary Figure S3new.tif]

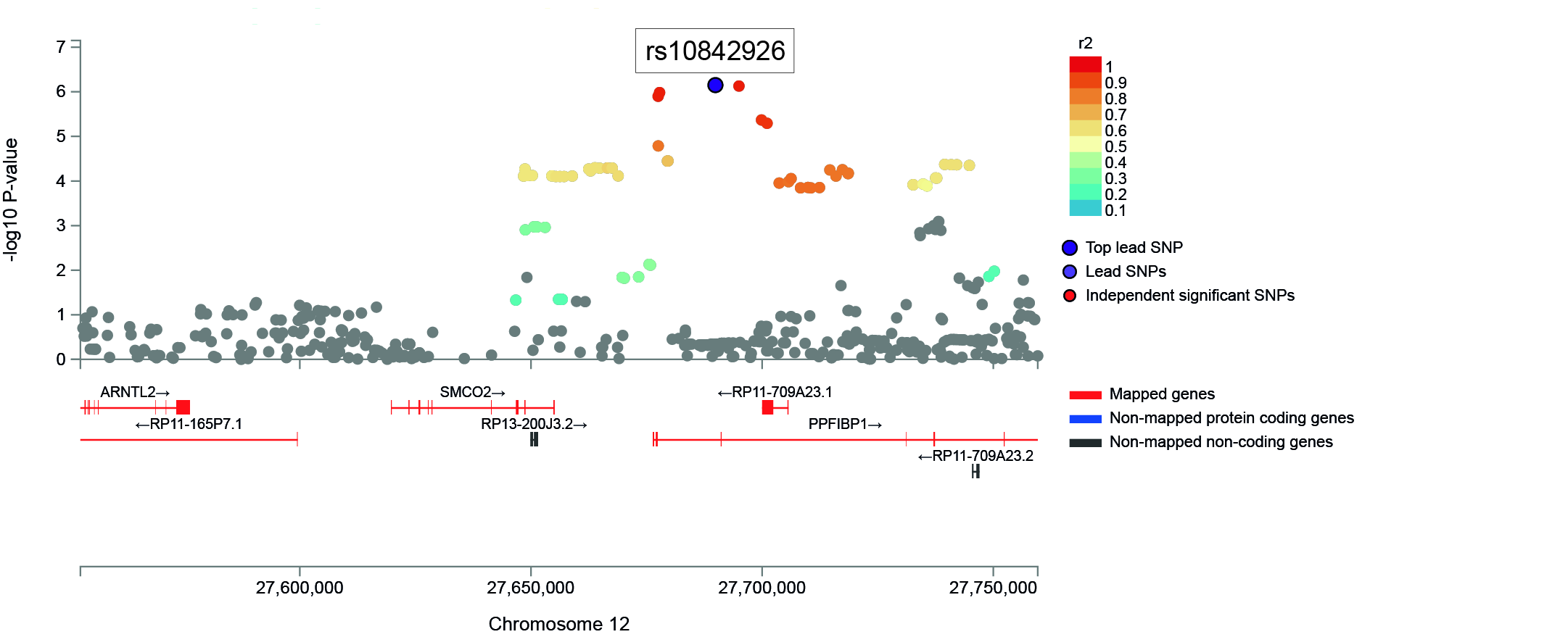

Supplement: Supplementary file 1 [file ijms-24-14844-s001.zip › Supplementary figure S4new.tif]

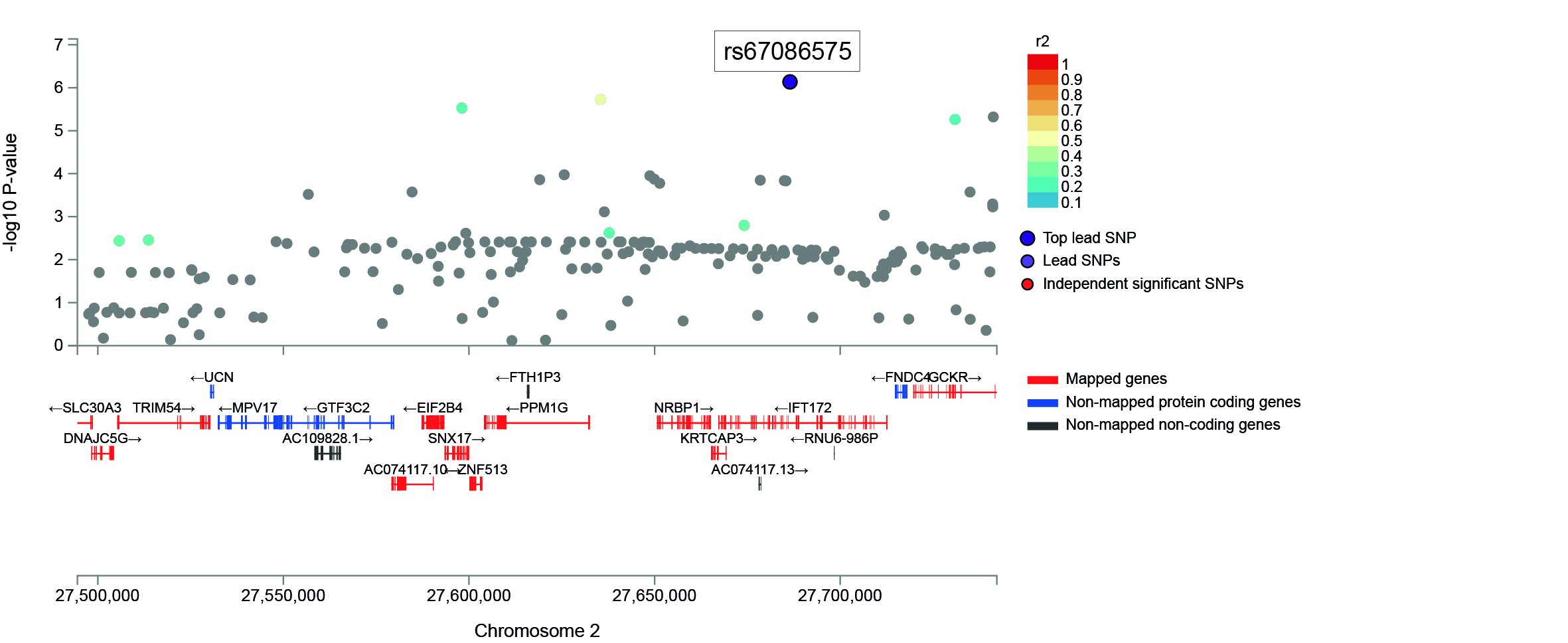

Supplement: Supplementary file 1 [file ijms-24-14844-s001.zip › Supplementary figure S5new.tif]
